# Mindfulness Training Improves Attention in Veterans with MCI and Mild Dementia: A Pilot Study

**DOI:** 10.1093/geroni/igaf122.3624

**Published:** 2025-12-31

**Authors:** Grace Desmond, Heera Kamaraj, Yasemin Yilmaz, Brenna Hagan, Kathy Xie, Katherine Turk

**Affiliations:** University of Massachusetts Chan Medical School, Worcester, Massachusetts, United States; VA Boston Healthcare System, Boston, Massachusetts, United States; VA Boston Healthcare System, Boston, Massachusetts, United States; VA Boston Healthcare System, Boston, Massachusetts, United States; VA Boston Healthcare System, Boston, Massachusetts, United States; VA Boston Healthcare System, Boston, Massachusetts, United States

## Abstract

Mild cognitive impairment (MCI) due to Alzheimer’s disease (AD) is often marked by early and progressive deficits in attention. Mindfulness—defined as focused, nonjudgmental awareness of the present moment—has been shown to improve attention and induce neuroplastic changes in healthy older adults. This pilot study examined whether a 10-week mindfulness intervention could improve attention in individuals with MCI. Participants were recruited from the VA Boston Memory Disorders Clinic and pseudo-randomly assigned to a virtual mindfulness-based stress reduction (MBSR) class (n = 9, mean age = 78.4 ± 2.06) or a waitlist control group (n = 11, mean age = 78.4 ± 4.76). The MBSR group received weekly group instruction, engaged in focused attention and open monitoring practices, and completed daily meditation via the Happier app. Attention was assessed at baseline and post-intervention using the Digit Span and Symbol Coding subtests from the Repeatable Battery for the Assessment of Neuropsychological Status (RBANS), and the AX-Continuous Performance Task (AX-CPT). Compared to the control group, MBSR participants showed significantly greater improvement in Digit Span (F(1,18) = 10.71, p = 0.004, η² = 0.37) and AX-CPT D’ scores (F(1,18) = 6.73, p = 0.018, η² = 0.27), suggesting enhanced attention processes. While group differences in Symbol Coding and CPT reaction time and accuracy were not statistically significant, this study provides preliminary evidence that mindfulness may improve attentional functioning in Veterans with MCI. Ongoing work includes examining event-related potential (ERP) components (e.g., N200, P300) to clarify underlying mechanisms of attention change.

